# Evaluation of the Quick Wee method of inducing faster clean catch urine collection in pre-continent infants: a randomized controlled trial

**DOI:** 10.1007/s12519-021-00483-4

**Published:** 2021-11-19

**Authors:** Aoife Branagan, Niofa Canty, Evanne O’Halloran, Marian Madden, Michael Brendan O’Neill

**Affiliations:** Department of Paediatrics, Mayo University Hospital, Castlebar, Co. Mayo Ireland

**Keywords:** Infant, Urinalysis, Urinary tract infection, Urine specimen collection

## Abstract

**Introduction:**

The acquisition of non-contaminated urine samples in pre-continent infants remains a challenge. The Quick Wee method uses bladder stimulation to induce voiding. A previous randomized trial showed a higher rate of voiding within 5 minutes using this method. We evaluated this method in an Irish hospital providing secondary care.

**Methods:**

A non-blinded, randomized, controlled trial was carried out. Eligible infants were between 1 and 12 months of age, who required urine sampling as part of clinical care. Participants were randomly allocated to receive the intervention (Quick Wee Method—supra-pubic stimulation with cold saline) or the control (usual care—clean catch with no bladder stimulation) for 5 min. Primary outcome was voiding of urine within 5 min.

**Results:**

A total of 140 infants were included in this study (73 in intervention group; 67 in control group). Baseline characteristics were similar. 25% in the intervention group passed urine in the 5-min trial period compared with 18% in the control group [*P* = 0.4, absolute difference 7% (95% confidence interval: − 7% to + 20%)].

**Conclusion:**

The Quick Wee method is a simple and inexpensive intervention that did not show a statistically significant increase in urine samples obtained in pre-continent infants.

## Introduction

Urinalysis and urine culture are both integral to the assessment of infants who are febrile or present with acute undifferentiated symptom complexes. The American Academy of Pediatrics (AAP) recommends invasive procedures, such as urethral catheterisation or suprapubic aspiration, to obtain urine samples in precontinent infants [1]. A study by Selekman based on the experience of 2726 parents of infants who underwent urinary catheterization reported that 56% of parents were extremely distressed by the procedure [2].

The NICE guidelines, which were updated in 2018, recommend a clean catch strategy for urine collection in pre-continent infants and children [3]. The time taken to obtain a clean catch urine sample can be considerable. In a study by Tosif of children aged 2–48 months, 139 (64%) had a successful clean catch urine specimen obtained; however, the median time taken was 30.5 minutes [interquartile range (IQR): 11–61 minutes] [4]. The guidelines also actively discourage the use of bag specimens to obtain urine samples.

Recently, the Quick Wee method of bladder stimulation, which uses a non-invasive strategy to induce voiding within 5 minutes, was evaluated by Kaufman in a tertiary care centre. In this evaluation, the Quick Wee method was an effective method for obtaining clean catch urine specimens in infants 1–12 months of age with the primary outcome being achieved in 31% of the intervention group versus 12% of the control group [*P* < 0.0001, absolute difference 19%, number needed to treat = 5 (NNT)] [5].

Prior to undertaking the present study, we utilized urine bags in precontinent infants to obtain urine samples because it is customary and a matter of practice not to use invasive procedures routinely to obtain urine in Ireland. Bag specimen urine collection had been well established in our unit and was seen as both convenient and time-efficient by the nursing staff. The Quick Wee method, if effective would allow our practice to be modified in keeping with current NICE guidelines. We therefore undertook a replication study of the Quick Wee method of urine collection in our secondary care institution to evaluate the effectiveness of this method.

## Methods

Ethical approval was obtained from the Research Ethics Committee of Mayo University Hospital, Ireland prior to initiation of the trial (REC approval number 20181001). The trial was registered with the ICTRN registry (ISRCTN43796385).

We performed a prospective, randomized, non-blinded replication trial between February and July 2019 in both the Emergency Department and Pediatric Decision Unit of Mayo University Hospital, which provides secondary pediatric care with 7500 emergency paediatric presentations per year.

### Participants

Infants aged 1–12 months, from whom a urine sample was required as part of their clinical care, were eligible for inclusion if parental informed consent was obtained.

Exclusion criteria were as follows: (1) infants with anatomical abnormalities affecting voiding; (2) clinically unwell infants requiring immediate treatment as determined by the treating clinician, and (3) an inability to obtain informed consent from parents. Data collected included: (1) demographic details (age, sex, clinical co-morbidities); (2) clinical details inclusive of previous UTI; (3) an assessment of level of dehydration, based on a 5-point Likert scale, ranging from none to severe; (4) the achievement of the primary outcome, and (5) the achievement of secondary outcomes.

### Intervention

Infants were randomized to standard care or standard care plus the Quick Wee intervention. Standard care consisted of cleaning the perineum with clean gauze soaked with sterile water at room temperature for 10 seconds and waiting for 5 minutes in anticipation of the infant voiding. The Quick Wee intervention consisted of rubbing the supra-pubic area with gauze soaked with sterile saline. Viles of saline solution were stored in a refrigerator at 2.8 °C and were used to soak clean gauze just prior to commencing the intervention. The gauze soaked with cold saline solution was used to rub the supra-pubic area for 5 min while a second examiner held a container in anticipation of the infant voiding. Stimulation with saline soaked gauze was performed for the duration of the 5-min study period using between 10 and 20 mL of cold saline. A screening dipstick urinalysis was performed on each collected urine sample and only if positive for leucocytes, nitrites or blood was the sample was sent for urine microscopy and culture. Contamination of a sample was defined as greater than 100,000 colony-forming units per mL of a mixed growth of organisms. If no voiding occurred within the 5-minute study period, the treating clinician decided either to wait for the infant to void, to apply a urine bag, or to abandon the procedure. A successful outcome was the passage of at least 1 mL of urine within the 5-minute time frame.

Training of the medical and nursing staff in the Quick Wee intervention was undertaken by the principal investigator, utilizing a didactic education module in conjunction with an educational pack. The principal investigator was in attendance to ensure compliance with the intervention strategy. Parents could perform each of the three tasks involved (standard cleaning, intervention and catching of sample) under the supervision of a staff member who had undergone training. For each infant enrolled in the study, standard equipment and Quick Wee equipment were prepared at the bedside before the opening of the envelope containing the group assignment and prior to the opening of the infant’s nappy.

### Outcome

The primary outcome of this study was the passage of 1 mL of urine within 5 min of the application of standard care versus the application of standard care in conjunction with the Quick Wee intervention.

The secondary outcome measures recorded were: (1) the time taken to void; (2) the contamination rates of laboratory assessed urine sample, and (3) the parental and clinician satisfaction rating based on a 5-point Likert scale ranging from 1 (very unsatisfied) to 5 (very satisfied).

### Sample size calculation

A power calculation was performed prior to the initiation of the study. Given the difference of 19% between groups found in the original study, a difference of 20% between the rate of acquisition of urine samples between the intervention and control group was utilized. This clinically important difference would enhance the transition of urine collection from bag specimen to clean catch urine collection. To detect a difference of 20% in the rate of acquisition of urine samples between intervention and control groups with 80% power and 5% significance, 62 patients would be required in each group, 124 patients in total.

### Randomization and allocation concealment

Participants were randomly allocated to the intervention (Quick Wee) group or control (standard care) group utilizing random permuted blocks of eight. Allocations were concealed in sealed opaque envelopes, within individual study packs. Randomization was performed by shuffling sealed opaque envelopes. Due to the nature of the intervention, blinding was not possible.

### Patient and public involvement

No patient/public involvement was sought to assist in the design of the research question or study structure because the present study was a replication study. Patient representatives were involved in the ethics committee who approved the study at our institution. The potential burden of the intervention was not assessed by the patients, but parental satisfaction with the method was assessed utilizing a Likert scale.

### Statistical analysis

Data were collected on paper record forms and were entered into an Excel spreadsheet. An Excel (Microsoft, USA) spreadsheet was used to collate data. Statistical analysis was performed using GraphPad QuickCalcs (accessed between September 2019 and September 2021) [6]. Baseline characteristics of the cohort were summarized using mean, standard deviation, median, interquartile range (IQR), and percentage as appropriate. *P* values were calculated using a two-tailed Fisher’s exact test and student *t*-test where applicable. *P* values < 0.05 were considered statistically significant.

## Results

**Fig. 1:**
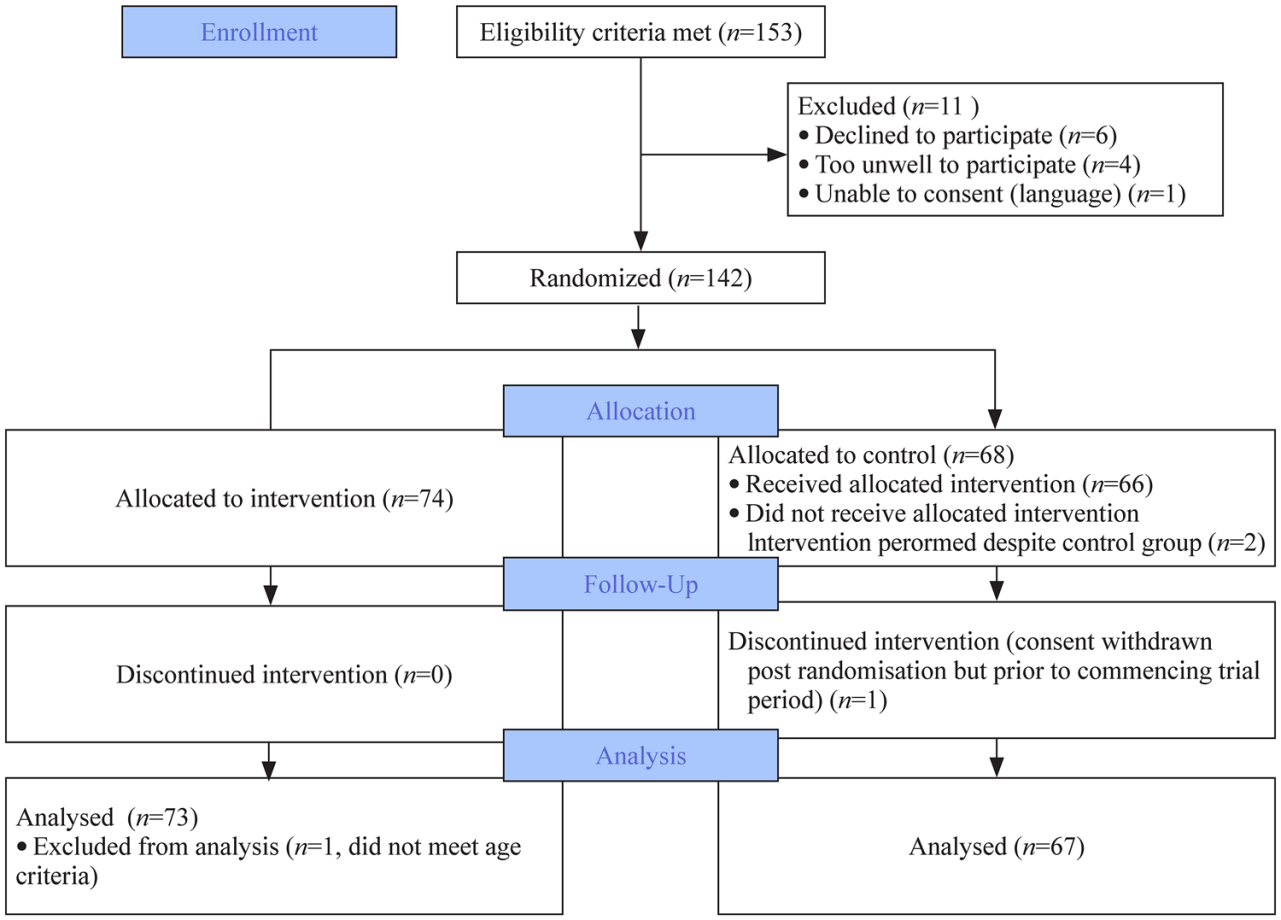
Consort flow diagram

Patient flow through the study is detailed in Fig. 1. The parents of 153 infants were approached to take part in the study, and 142 parents agreed. 140 infants were included in an intention-to-treat analysis, with 73 in the intervention group and 67 in the control group. One infant in the intervention group was excluded prior to analysis because the age criterion was breeched. There was one withdrawal post-randomization prior to starting the trial period in the control group. Two infants were randomized to the control group but had the intervention performed due to human error. These were included in the intention-to-treat analysis.

The baseline characteristics were similar in both groups. These are detailed in Table 1. Comorbidities of included infants were recorded by enrolling nurse/doctor. Intervention group: laryngomalacia (*n* = 1), mild hydronephrosis (*n* = 1), hydrocephalus with ventriculoperitoneal shunt (*n* = 2); Control group: prematurity (*n* = 1), hydrocephalus with ventriculoperitoneal shunt (*n* = 2), vaccinations in prior 24 h (*n* = 1), developmental dysplasia of hip (*n* = 1), trisomy 21 (*n* = 1). The clinical indications for urine collection are outlined in Table 2. Clinicians could list more than one indication.

**Table 1:** Demographic and clinical characteristics of participants

Variables	Intervention % (*n*)	Control % (*n*)	*P* value
Male	54.8 (40)	61.2 (41)	0.5
Mean (SD) age in months	5.36 (3.58)	5.1 (3.27)	0.66
Age under 6 months	60.27 (44)	71.64 (48)	0.21
Recruited by			
Medical	76.7 (56)	85.1 (57)	0.28
Nursing	23.3 (17)	14.9 (10)	0.28
Co-morbidities			
Yes	5.5 (4)	8.9 (6)	0.52
No	94.5 (69)	91.4 (61)	0.52
Previous UTI			
Yes	4.1 (3)	1.5 (1)	0.62
No	94.5 (69)	97 (65)	0.68
Unknown	1.4 (1)	1.5 (1)	1.0
Antibiotics previous 24 h			
Yes	19.2 (14)	9 (6)	0.1
No	80.8 (59)	91 (61)	0.1
Hydration			
Normal	80.8 (59)	77.6 (52)	0.68
Mild	15.1 (11)	20.8 (13)	0.51
Moderate	4.1 (3)	1.5 (1)	0.62
Severe	0 (0)	1.5 (1)	0.48
Likelihood UTI			
No possibility	15.1 (11)	13.4 (9)	0.81
Very unlikely	68.5 (50)	74.6 (50)	0.46
Quite unlikely	13.7 (10)	11.9 (8)	0.81
Very likely	2.7 (2)	0	0.5
Certain	0	0	1.0

*UTI* urinary tract infection. Values are percentage (*n*) unless otherwise stated

**Table 2:** Indications for sample and clinical signs at presentation

Variables	Intervention group % (n)	Control group % (n)
Indication for samples:		
a) Unsettled baby	56.1 (41)	63.2 (36)
b) Fever unknown origin	21.9 (16)	16 (11)
c) Poor feeding	17.8 (13)	25.4 (17)
d) Other	17.8 (13)	16.4 (11)
e) Suspect UTI likely	5.5 (4)	3 (2)
f) Failure to thrive	1.4 (1)	3 (2)
g) Metabolic test	0 (0)	1 (1)

All values presented are percentage (*n*)

In the study, recruitment was performed by 113 (81%) doctors and 27 (19%) nursing staff. Standard cleaning was performed by 91 (65%) doctors, 38 (27%) nursing staff, and 11 (8%) parents. Catching of urine samples in a sterile container was performed by 44 (32%) doctors, 31 (22%) nursing staff, and 65 (46%) parents. The intervention, rubbing of cold saline to the suprapubic area, was performed by 49 (67%) doctors, 19 (26%) nursing staff, and 5 (7%) parents.

The primary outcome was achieved in 18 (25%) of the intervention group versus 12 (18%) in the control group[*P* =0.4, absolute difference: 7%, 95% confidence interval (CI): − 7% to + 20%, NNT: 14]. Overall, 30 (21%) infants passed urine within 5 minutes. Secondary outcomes recorded were: (1) the time to void; (2) the contamination rate of urine sample with abnormal urinalyses, and (3) the parental and clinician satisfaction on a 5-point Likert scale.

In the intervention group, the mean time to void was 121 seconds [standard deviation (SD) 97 seconds], compared with 150 seconds (SD 115 seconds) in the control group (*P*=0.47). The mean difference was 29 seconds (95% CI: − 108 to 51). Although 30 infants voided within 5 minutes, there was a failure to collect 1 sample and consequently only 29 urine samples were collected, of which 12 (41%) had negative urinalysis. Seventeen (59%) samples had an abnormal urinalysis (defined as dipstick stick testing positive for any of the following: leukocyte esterase, nitrites, or blood, protein or any combination thereof) and were sent for urine microscopy and culture.

Of the 17 samples sent for culture, 1 sample had > 100 white cell count (wcc) and pure growth of *Escherichia coli*, 7 had negative microscopy and culture (0 wcc and no growth of organisms), 6 had < 20 wcc and 10,000–50,000 CFU of mixed growth of bacterial organisms, 1 had < 20 wcc and 50,000–100,000 CFU of mixed growth of bacterial organisms and 2 met the definition of contamination, < 20 wcc on microscopy with greater than 100,000 CFU of mixed growth of bacterial organisms.

Urine samples containing mixed bacterial growth on culture were equal between control and intervention group, 54% (6 of 11) in the intervention group compared with 60% (3 of 5) in the control group. Overall, nine (31%) of urines collected in the study were contaminated.

**Fig. 2:**
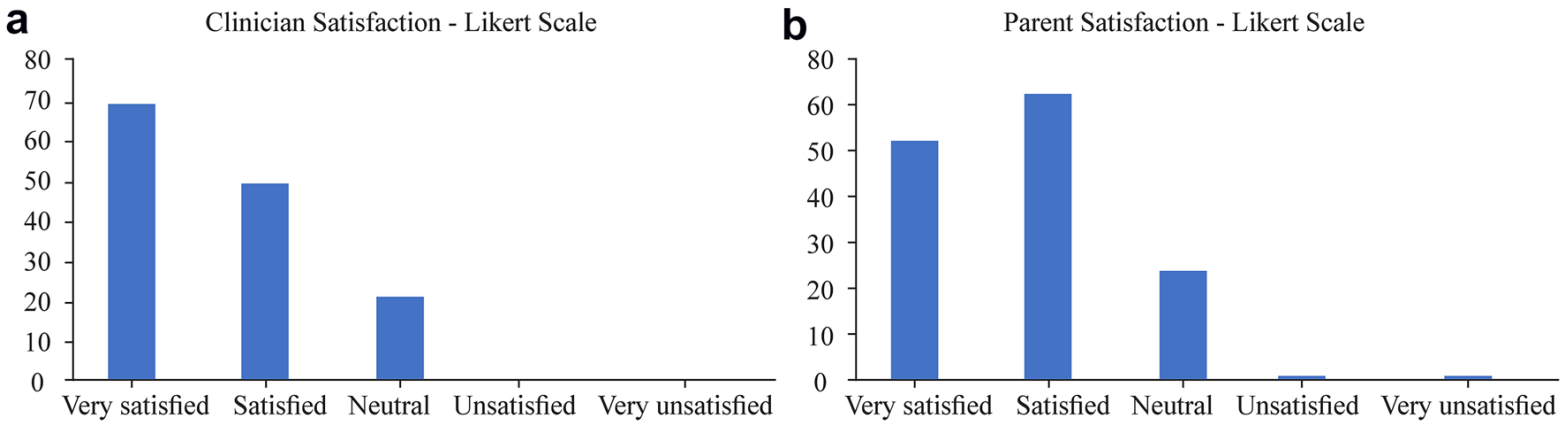
Likert score of satisfaction recorded by clinicians (**a**) and parents (**b**)

The study was accepted well by both parents and clinical staff. There were no significant difficulties reported in either group. On a five-point Likert scale (very unsatisfied to very satisfied), the mean score for parents was 4.16 with a positive skew (percentage of respondents choosing point 4 or 5 of scale) of 81% and mean score for clinicians was 4.35 with a positive skew of 83.6% (Fig. 2).

We undertook a post hoc analysis based on age. A total of 92 infants were included in the group between 1 and 6 months, including 44 in the intervention group and 48 in the control group. Twelve of the 44 infants (27%) in intervention group and 11 of 48 (23%) in the control group passed urine in the 5-min intervention period. The absolute difference was 4%, and 95% confidence intervals: 13.4% to 22.1% (*P* = 0.8). The number needed to treat to obtain one extra sample in this cohort was 23.

In the group older than 6 months of age, there was a total of 48 infants, including 29 in the intervention group and 19 in the control group. Six infants in the intervention group (21%) compared to one infant (5%) in the control group voided. The absolute difference was 16%, 95% confidence interval, 2.4% to 33.3% (*P* = 0.25). The number needed to treat in this cohort was 7.

## Discussion

The present study was a replication study of the Quick Wee method of urine collection in pre-continent infants from 1 month to 1 year of age and was carried out in a hospital providing secondary care [5]. We did not observe a statistically significant difference in urine collection obtained using this method. The success rate was 18% in the control group and 25% in the Quick Wee group (Absolute difference 7%, 95% CI − 7% to 20%, NNT 14). In the original study by Kauffman, the success rate was 12% in the control group and 31% in the Quick Wee group.

To date and to our knowledge, there have not been other studies registered to evaluate the Quick Wee method against standard methods of urine collection. It is possible that non-registered trials have been performed that did not show statistically significant results. Such studies are less likely to be published [7–9].

Failure to replicate the results of original trials has been shown across many areas of medicine. Ioannidis et al. showed that 44% of highly cited studies had results inconsistent with a replication attempt [10]. The author cites differences in the disease spectrum, population, eligibility criteria, and the use of concomitant interventions as potential explanations.

The Quick Wee method is based on the physiology of micturition in young infants. Stimulation of the voiding reflex can be utilized to obtain urine samples prior to the development of the control of voiding, which normally develops at approximately 2 years of age via the pudendal nerve [11]. This physiological feature can be provoked by stimulating the bladder or perineal skin [11, 12]. Cold stimulation will trigger parasympathetic detrusor contraction through the somato-bladder reflex mechanism and can similarly be utilized [12–14]. This reflex may be temperature sensitive. The temperature in Castlebar during the 5-month study period ranged from 5 to 15 °C compared to Melbourne which ranged from 15 to 21 °C during a 10-month study period. The level of dehydration or illness in the cohorts in our study was similar to those in the study by Kaufman.

Parents could undertake specific tasks in this study. 8% (11) of parents undertook standard cleaning, 7% (5) performed the suprapubic rubbing with cold saline, with two infants voiding, and 46% (65) were assigned to urine collection. This active engagement may have contributed to the high parent satisfaction rating, and it reflects current pediatric practice of partnership with parents in the care of their children. Parental involvement, especially in urine collection, is essential in clinical practice.

In our study, the impact of specific potential influencers included: (1) the interval from the infant’s last void; (2) the impact of feeding directly before the collection of urine, and (3) the infant’s age were not evaluated. The frequency of voiding in infants is quoted in the literature as four times per 4-h period up to 6 months of age and three times per 4-h period for ages 6–12 months [11, 14]. It is plausible that a longer time period since the infant’s last urine void and the administration of fluid (e.g., 20 mL/kg) would be expected to result in more urine being present in the bladder. This could be assessed utilizing point-of-care ultrasonography. This should then theoretically result in reflex emptying on application of the Quick Wee method. These factors need to be assessed in future studies.

A post hoc subgroup analysis explored differences in utility of the method based on age, given the changing physiology of infant micturition over the first year of life. We saw a larger positive response to this method in the group over 6 months (absolute difference of 4% with NNT of 23 in the 1–6-month group compared with a 16% absolute difference with number needed to treat of 7 in the over 6-month group). These results must be interpreted with significant caution because we did not power the study sufficiently for this outcome and because the absence of stratification based on age prior to randomization led to a significant difference in group size. In our study and in Kaufmann’s, the percentage of patients had not voided at 5 minutes were similar (75% and 69%)[5].

Our current practice is to actively encourage waiting, until the infant voids spontaneously, because it is customary and common practice not to routinely perform infantile catheterization. We actively dissuade the use of bag specimens for urine collection. Other non-invasive strategies can be used to obtain urine samples from pre-continent infants, as reported by Herreros Fernandez, et al [15]; however, the strategy relies on a combination of specific fluid intake and non-invasive bladder stimulation [15]. This process consists of the following steps: (1) the administration of 25 mL/kg of fluid, if the infant is older than 2 weeks; (2) 25 minutes post feeding, the genitals are cleaned thoroughly with warm water and soap and dried with sterile gauze; (3) prior to commencing the non-invasive bladder stimulation the infant receives non-pharmacological analgesia, such as 2% sucrose or non-nutritive sucking to prevent or reduce crying, and (4) the infant is held under the armpit with the legs dangling and tapping of the suprapubic area at a frequency of 100 taps per minute for 30 seconds, followed by lumbar stimulation manoeuvres (light circular massage in the lumbar paravertebral region for 30 seconds) commences. This process is continued for 5 minutes, or until the infant voids. This method requires specific training to ensure competence and requires non-pharmacological analgesia to reduce infant distress levels. The original cohort study involved 80 infants under 1 month of age, and 69 (86.3%) infants voided within 5 minutes.

This study was replicated by Crombie in a cohort of 147 infants < 90 days old [16]. The success rate of induced voiding was lower at 53.1%. Both studies utilized fluid administration and bladder stimulation but the relative contributions of these interventions were not assessed. Another study by Tran modified the bladder and lumbar stimulation protocol in non-ambulant infants. Children < 2 years were recruited, and the stimulation time was reduced to 3 min [17]. If voiding did not occur, infants had oral fluids administered prior to a second attempt. Of the inception cohort of 142 patients, 60 (42.2%) voided within 3 min. Of the 82 who received fluids and had a second episode of stimulation, 19 (23.1%) infants voided. A key finding of this study was the impact of age on the primary outcome. Of the 27 infants < 1 month old, 24 (88.9%) voided as opposed to 2 (28.6%) of the 7 participants > 1 year of age. Cohort studies can overestimate effect sizes and consequently, an RCT by Demoncy to define the impact of bladder stimulation, as a non-invasive technique to collect urine, to diagnose UTI in infants < 6 months is currently underway [18].

The main strength of our study was adherence to the core elements of an RCT with true randomization and allocation concealment. Our study was adequately powered to detect the 20% clinical difference that we sought to modify our clinical practice. We used a simple technique, which is not resource-intensive when compared to the bladder and lumbar stimulation technique described above and used in other studies, to address a common pediatric challenge. The high levels of acceptability of this method will aid practice modification.

Limitations of this study include: (1) not stratifying infants by age, i.e., 1–6 months and > 6 months; and (2) not determining the time of the last void by infants prior to study enrolment.

In conclusion, this prospective, randomized, non-blinded superiority trial did not show a statistically significant benefit for carrying out the Quick Wee method of inducing urine collection in pre-continent infants.

## Data Availability

The datasets generated during and/or analysed during the current study are available from data repository—https://figshare.com/articles/dataset/Data_repositoryQW_xlsx/16571265
